# Ursolic Acid-Regulated Energy Metabolism—Reliever or Propeller of Ultraviolet-Induced Oxidative Stress and DNA Damage?

**DOI:** 10.3390/proteomes2030399

**Published:** 2014-08-06

**Authors:** Yuan-Hao Lee, Youping Sun, Randolph D. Glickman

**Affiliations:** 1 Department of Oncologic Sciences, Mitchell Cancer Institute, University of South Alabama, Mobile, AL 36604, USA; 2 Department of Radiation Oncology, Center for Radiological Research, Columbia University Medical Center, New York, NY 10032, USA; E-Mail: ys2611@columbia.edu; 3 Department of Ophthalmology, Center for Biomedical Neuroscience, The University of Texas Health Science Center at San Antonio, San Antonio, TX 78229, USA; E-Mail: glickman@uthscsa.edu

**Keywords:** ultraviolet, phosphatidylinositol 3-kinase-related kinases, hormone receptors, ursolic acid

## Abstract

Ultraviolet (UV) light is a leading cause of diseases, such as skin cancers and cataracts. A main process mediating UV-induced pathogenesis is the production of reactive oxygen species (ROS). Excessive ROS levels induce the formation of DNA adducts (e.g., pyrimidine dimers) and result in stalled DNA replication forks. In addition, ROS promotes phosphorylation of tyrosine kinase-coupled hormone receptors and alters downstream energy metabolism. With respect to the risk of UV-induced photocarcinogenesis and photodamage, the antitumoral and antioxidant functions of natural compounds become important for reducing UV-induced adverse effects. One important question in the field is what determines the differential sensitivity of various types of cells to UV light and how exogenous molecules, such as phytochemicals, protect normal cells from UV-inflicted damage while potentiating tumor cell death, presumably via interaction with intracellular target molecules and signaling pathways. Several endogenous molecules have emerged as possible players mediating UV-triggered DNA damage responses. Specifically, UV activates the PIKK (phosphatidylinositol 3-kinase-related kinase) family members, which include DNA-PKcs, ATM (ataxia telangiectasia mutated) and mTOR (mammalian target of rapamycin), whose signaling can be affected by energy metabolism; however, it remains unclear to what extent the activation of hormone receptors regulates PIKKs and whether this crosstalk occurs in all types of cells in response to UV. This review focuses on proteomic descriptions of the relationships between cellular photosensitivity and the phenotypic expression of the insulin/insulin-like growth receptor. It covers the cAMP-dependent pathways, which have recently been shown to regulate the DNA repair machinery through interactions with the PIKK family members. Finally, this review provides a strategic illustration of how UV-induced mitogenic activity is modulated by the insulin sensitizer, ursolic acid (UA), which results in the metabolic adaptation of normal cells against UV-induced ROS, and the metabolic switch of tumor cells subject to UV-induced damage. The multifaceted natural compound, UA, specifically inhibits photo-oxidative DNA damage in retinal pigment epithelial cells while enhancing that in skin melanoma. Considering the UA-mediated differential effects on cell bioenergetics, this article reviews the disparities in glucose metabolism between tumor and normal cells, along with (peroxisome proliferator-activated receptor-γ coactivator 1α)-dependent mitochondrial metabolism and redox (reduction-oxidation) control to demonstrate UA-induced synthetic lethality in tumor cells.

## 1. Background

### 1.1. Effects of UV in Sunlight

The majority of solar radiation delivered to Earth surface is distributed in the region of near-infrared, accompanied by far ultraviolet (UV) radiation and visible light. This broadband radiation, although containing only a small fraction of the UV radiation, has the potential to cause skin damage and malignant transformation [1,2,3]. The spectrum of ultraviolet light is divided up into UVA (315–400 nm), UVB (280–315 nm) and UVC (200–280 nm) [4]. The UVC is completely absorbed by the atmosphere whereas the UVB is only partly absorbed and the UVA is not. UV radiation of energy greater than 6 eV can ionize water molecules into hydroxide ions and protons that enable photochemical reactions within cells whereby endogenous chromophores can be excited to undergo the Type I and II reactions [4,5,6]. Singlet oxygen generated from endogenous chromophores can not only result in DNA interstrand cross-linking but also cause photochemical modification of nitrogenous bases [7]. The oxidization of guanosine residues into 7-hydro-8-oxodeoxyguanosine leads to guanine-thymine transversion mutation [8]. Because the aromatic ring structures of purine and pyrimidine bases are strong absorbents of light with wavelengths in the range of 230–300 nm, direct photodamage of DNA can be readily caused by UVB*.* Adjacent pyrimidines are subject to dimerization upon UVB irradiation, and the resultant cyclobutane pyrimidine dimers (CPDs) may result in cytosine-thymine base transitions in case of insufficient DNA repair [9]. In contrast, UVA indirectly causes CPDs and (6-4) photoproducts by producing free radicals [10,11]. Under equimutagenic doses, UVA induces higher rates of mutation formation at DNA photoproducts than UVB does due to its antagonistic effect on cellular DNA damage responses. In addition, the oxidation-reduction (redox) reaction of plasma membrane electron transport systems, but not that of mitochondrial electron transport chain, is affected by UVA for inducing light toxicity [12]. Considering that sunlight is a broadband radiation, the interplay of multiple spectrum wavelengths may lead to different cellular responses. One of the important light effects that decelerate mutagenesis is through activation of tyrosine kinases, such as insulin and insulin-like growth factor 1 (IGF-1) receptors [13]. Researchers have found that (IGF-1)-mediated AKT activation delays UVB-induced apoptosis, allowing more time for removing cyclobutane thymine dimers in primary human keratinocytes [14]. Additionally, IGF-1 activation in mammalian and rat cell lines was found to facilitate homologous recombination repair by mediating IRS-1 (insulin receptor substrate 1) phosphorylation and promoting Rad51 trafficking to the site of damaged DNA [13]. In photosensitive tissues, such as retinal rod outer segments, intrinsic tyrosine kinases can be activated by light [15]. Bell* et al.* have reported that a 97-kDa protein (later known as β-subunit of the insulin receptor) in retinal rod outer segments is actively phosphorylated* in vitro* under conditions that favor tyrosine phosphorylation [16]. The 97-kDa protein was also found by Ghalayini* et al.* to be phosphorylated in rat retinal rod outer segments in a light-dependent manner [17]. This indicates that activation of insulin receptor generally reduces cellular photosensitivity by counteracting UV-induced pro-apoptotic cell signaling [18]. By applying broadband radiation from a mercury arc lamp, the light-induced p53 and NF-κB activation of retinal pigment epithelial (RPE) were both observed to be enhanced by the pretreatment with insulin and the insulin receptor sensitizer, ursolic acid (UA) [18]. 

### 1.2. Ursolic Acid and Its Biological Functions

#### 1.2.1. Antioxidant Activity

**Figure 1:**
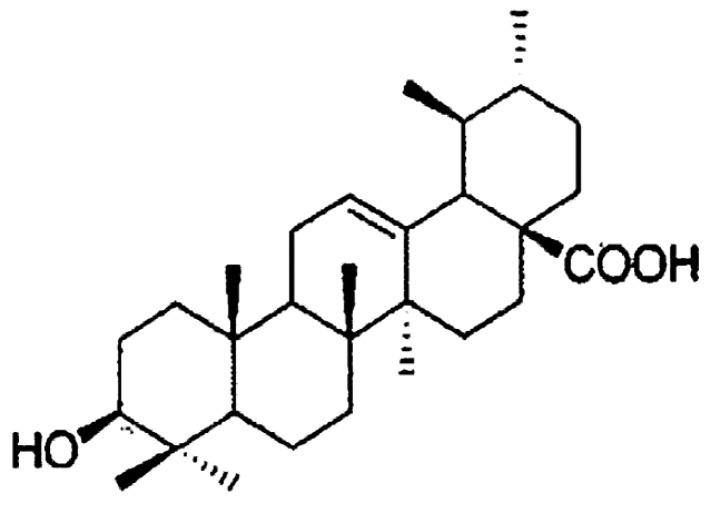
Structural formula of UA. UA is also called prunol, malol, *β*-ursolic acid, NSC4060, CCRIS 7123, TOS-BB-0966, 3-*β-*hydroxyurs-12-en-28-oic acid with a molecular formula of C_30_H_48_O_3_ and a molar mass of 456.7 g/mol. Adaped with permission from [25].

UA (structure shown in Figure 1) is a naturally occurring triterpenoid compound present in a wide variety of fruits and vegetables, including basil, apples, and cranberries. UA exhibits antibacterial activity against plant pathogens and is reported to have a range of actions in cells and tissues. Studies have shown that UA can ameliorate oxidative damage via free radical scavenging and enzymatic activity modulation. For instance, UA was found to inhibit the activity of lipoxygenase in murine macrophages, human platelets and HL60 leukemic cells and reduce the production of leukotrienes [19]. Additionally, UA has been found to possess antioxidant activity that pharmacologically modifies human enzymes, including superoxide dismutase, catalase, glutathione reductase, glutathione peroxidase, and glutathione levels in the liver [20]. Furthermore, UA decreases hepatotoxicity by slowing the clearance of chemotherapy drugs via cytochrome P-450 enzymes in the liver and decreases H_2_O_2_ production via the uncoupling of mitochondrial oxidative phosphorylation in the heart [21,22]. This antioxidant effect correlates with the reduced UV-induced lethality found in healthy cells treated with UA [23,24]. Thus, UA is postulated to confer photoprotection on normal cells.

#### 1.2.2. Capabilities of Ceramide Stabilization and Surface Protein Recognition

Lipid peroxidation is strongly induced by UV [26,27]. Among various kinds of natural compounds, triterpenoids are lipophilic molecules that can confer photoprotection on UV-exposed cells through stabilization of lipid rafts [28]. Ursolic acid and its isomer, oleanolic acid, have been found to tightly pack with sphingomyelin and glycosphingolipids due to the steric hindrance presented by their methyl groups. Due to the fact that UA stabilizes the ceramide structure, ceramide-mediated apoptosis can be reduced by UA upon irradiation. Given that the internalized surface membrane protein, CD36, acts as a mediator for the functions of fatty acid transporters, its association with UA may stimulate cell uptake of fatty acid and trigger sensitization of insulin receptors along with suppression of lipid synthesis [29,30,31].

#### 1.2.3. Antitumoral Activity

UA is found to be potent in cancer suppression with its cytostatic and cytotoxic activity in tumor cells. It exerts an early cytostatic effect at G1 followed by cell death through apoptosis. The pro-apoptotic effect of UA is associated with the up-regulation of bax and down-regulation of bcl-2 leading to mitochondria-mediated apoptosis [32,33]. Concomitantly, UA inhibits NF-κB (nuclear factor kappa-light-chain-enhancer of activated B cells) activation and mediates tumor cell apoptosis via activation of p53 and/or caspase-3 [32,33,34,35]. The antitumoral effect of UA exerted at high dosages is associated with inactivation of the PI3K (phosphoinositide 3-kinase)-AKT-mTOR (the mammalian target of rapamycin)-(NF-κB) signaling pathway, which is in turn activated for reducing endoplasmic reticulum stress and restores insulin signaling in mice fed with a high fat diet [35,36]. This indicates that the inhibitory effect of UA on glucose metabolism may selectively attenuate tumor resistance to UV by blocking cancer cells’ high glycolysis rate, which otherwise would raise antioxidant defenses through inhibition of pyruvate kinase M2 and increased entry into the pentose phosphate pathway (PPP) [37,38]. The increased PPP pathway activity and resultant production of GSH (reduced form of glutathione) and NADPH (reduced form of nicotinamide adenine dinucleotide phosphate) may lead to enhanced cellular antioxidant capacity [38]. In summary, UA exerts pharmacological actions that lead to radiation sensitization not only through induction of apoptosis but also perturbation of redox homeostasis in tumor cells.

## 2. UV-Induced Mitogenic Activation for Cell Survival

UV-induced photochemical reactions impair cellular redox homeostasis and lead to the oxidation of cellular constituents [18]. The oxidation of the catalytic cysteine residues of protein tyrosine phosphatases (PTPs) by UV results in the prolonged activation of tyrosine kinase-coupled receptors (TKRs) because of sustained phosphorylation [39,40]. UV-induced DNA damage responses also play a role in maintaining the activity of TKRs through endogenous signaling. Upon UV irradiation, p53 activation, mediated by DNA-PK (DNA-dependent protein kinase) and ATM (ataxia telangiectasia mutated), arrests cells in G1/S, intra S, and G2/M phases through up-regulation of p21^Cip1^ and the subsequent inhibition of CDK (cyclin-dependent kinase)-cyclin complexes and PCNA (proliferating cell nuclear antigen) [41,42,43]. The cell cycle arrest facilitates non-homologous end-joining (NHEJ) repair machinery due to the negative regulatory effect of p53 on Rad51 transcription via binding to the p53 response element of Rad51 promoter [44]. On the other hand, UV-induced p53 activation inhibits the MDM2 (mouse double minute 2 homolog)-mediated ubiquitination of IGF-1 (insulin-like growth factor 1) receptor by sequestering MDM2 to the nucleus [45]. The up-regulation and activation of IGF-1 receptor, in turn, facilitates the nuclear exclusion of p53, followed by MDM2-mediated lysosomal degradation [45]. The negative feedback loop maintains temporary activation of p53 for eliciting its effect on DNA repair, but not the protracted effect on cellular apoptosis. In 2001, Hѐron-Milhavet* et al.* showed that UV-mimetic-induced DNA damage by 4-nitroquinoline 1-oxide (4NQO) was reduced by activation of the IGF-1 receptor and p38 MAPK signaling pathway [46]. A year later, Hѐron-Milhavet and LeRoith published a study demonstrating that IGF-1 receptor signaling can rescue UV-mimetic-induced p53 up-regulation via the transcription of MDM2 and the dissociation of MDM2 from the nucleolar protein, p19^Arf^ [45,47]. These findings imply that p53-mediated DNA damage responses coordinates with activation of TKRs to sequentially down-regulate p53 activity for cell survival. 

In response to receptor tyrosine phosphorylation, the cytoplasmic association of insulin/IGF-1 receptor with insulin receptor substrates 1 and 2 (IRS-1/2) through the SH2 domain leads to the phosphorylation of IRS-1/2 by the β-subunit of insulin/IGF-1 receptors [48,49]. By functioning as a second messenger, IRS-1/2 interacts with and activates the regulatory subunits of PI3K and MAPK (mitogen-activated protein kinase) to effect a variety of cell activities, including cell proliferation, metabolism and apoptosis [49]. Following UV-induced TKR activation, phosphorylated IRS-1 and Shc bind to Grb-2 to trigger Ras-Raf-MEK (mitogen-activated protein kinase kinase)-MAPK signaling [50]. In addition, IRS-1 acts as an adaptor that facilitates PI3K activation through binding its pYMXM motif to the SH2 domains of p85 [48]. The resultant PI3K-AKT-(NF-κB) signaling cascade inhibits cell apoptosis induced by UV. Nevertheless, different cell lines respond to UV-induced TKR activation differently. Expression of the IGF-1 receptor on osteoblasts and fibroblasts was found in proportion to the UV-induced AKT activational phosphorylation by Thakur* et al.* [51]. Interestingly, those authors showed that UV-induced AKT phosphorylation of C2C12 myoblasts exclusively occurs under IGF-1 receptor deficiency. This controversial result indicates that cell specificity exists towards UV-induced TKR activation. 

### 2.1. UV-Induced Adaptive Defense against TKR-Mediated Mitogenic Effect

UVB was reported to desensitize insulin/(IGF-1)-mediated energy metabolism through down-regulation of the nuclear hormone receptors, PPARs (peroxisome proliferator-activated receptors) at the mRNA level. This occurs even though PPARγ can be activated by UVB via free radical-induced cleavage of endogenous glycerophosphocholines, leading to the expression of the pro-inflammatory proteins, COX-2 (cyclooxygenase-2) and prostaglandin E2 [52,53,54,55]. On the other hand, PPARγ is phosphorylated and inhibited by ROS-activated p38 MAPK, leading to decreased transactivation of genes that contain an evolutionarily conserved peroxisome proliferator response element consensus-binding site, including genes encoding enzymes involved in fatty acid oxidation and antioxidant defenses [53,56,57,58]. This regulation allows irradiated cells to prevent energy depletion via inhibition of UV-initiated mitochondrial oxidative stress [58]. The shutdown of mitochondrial metabolism, however, leads to insufficient energy production, resulting in an increase in the AMP (adenosine monophosphate)-to-ATP (adenosine triphosphate) ratio. As a result, the lack of ATP limits the conversion of ATP into cAMP (cyclic adenosine monophosphate) by UV-activated adenylyl cyclase. The increased AMP-to-cAMP ratio activates the glucose dependent G1-S checkpoint as well as p53 stabilization through AMPK (AMP-activated protein kinase)-mediated MDMX phosphorylation [59]. Stabilized p53 antagonizes glycolysis through activation of TIGER (TP53-induced glycolysis and apoptosis regulator) and inhibition of NF-κB-mediated GLUT3 (glucose transporter 3) gene transactivation. Through TIGER transactivation, p53 decreases the activity of phosphofructokinase 1 (PFK1) and increases the activity of fructose 2,6-biphosphatase (FBPase), leading to reduced glycolysis [33,60]. Additionally, p53 interacts with PTEN (phosphatase and tensine homolog) and negatively regulates AKT phosphorylation, resulting in a reduction of glycolysis upon mTOR inactivation [61]. As a downstream effecter of AKT, mTOR launches pro-survival signaling by coordinating metabolism with DNA repair. HIF-1α (hypoxia-inducible factor 1 alpha) is up-regulated by mTOR, thereby transactivating pyruvate kinase M2, whose RNA splicing is promoted by heterogeneous nuclear ribonucleoproteins (hnRNPs) upon mTOR activation [62,63]. In addition, expression of glucose transporters and the isozyme 1 of pyruvate dehydrogenase kinase (PDK1) are increased by mTOR-mediated HIF-1α (hypoxia-inducible factor 1α) translation, driving glycolysis for ATP generation in the absence of oxygen/mitochondrial respiration [64]. 

Taken together, cells that are less responsive to UV-induced mitogenic actions for energy production from glycolysis may rely on p53-mediated TIGER activation, which facilitates the pentose phosphate pathway (PPP) via increasing FBPase activity against UV-induced ROS [65]. In addition, we have observed oxidative stress in mitochondria increased by UV-VIS radiation (radiation with spectral wavelength ranging from ultraviolet to visible light, abbreviated as UVR hereafter), which, however, was decreased upon rapamycin pretreatment in retinal pigment epithelial cells, implying an intricate interplay between p53 and mTOR signaling in the regulation of mitochondrial metabolism [18].

### 2.2. UV-induced Cell Lethality through TKR-mediated Mitogenic Effects

From the point of view of energy metabolism, the degradation of one mole of palmitate can generate 138 moles ATP whereas a mole of glucose can generate 32 moles of ATP through oxidative phosphorylation following glycolysis [66,67]. Thus, UV-inhibited PPAR activity can reduce lipid oxidation and cause a marked decrease in intracellular ATP, leading to deficient cAMP signaling. The expression of many molecules involved in cell cycle regulation is regulated by cAMP via phosphorylation of the mediator, PKA (cAMP-dependent protein kinase). Studies have shown that Cdc20 (cell-division cycle protein 20) can be phosphorylated by the catalytic subunit of PKA, thus preventing Cdc20 from interacting with and mediating the proteolysis of two mitotic inhibitors, securin and Clb2 [68]. PKA also suppresses proteolysis of cyclin B and other factors that regulate sister chromatid separation via inhibitory phosphorylation of the ubiquitin ligase, anaphase-promoting complex/cyclosome (APC) [69]. Furthermore, the activity of Cdc25 phosphatase is maintained by PKA-dependent phosphorylation and therefore activates cdc2-cyclin B complex by dephosphorylation [70]. The identified mechanisms show how cAMP regulates the cellular responses to UV,* i.e.*, by suspending mitosis and explains the prevalence of UV-induced mitotic catastrophe as a consequence of the absence of PKA-induced G2 delay and mitotic arrest [71].

Furthermore, UV-induced IGF-1 activation induces anabolic biosynthesis instead of catabolic energy production, leading to the accumulation of intracellular AMP. Nevertheless, UV-induced AMPK activation can hardly transduce p53 activation in parallel with the IGF-1 signaling [72]. Transmitted by the (IGF-1)-PI3K-AKT pathway, MDM2 activity increases and mediates p53 ubiquitination and proteosomal degradation. Under UV-induced IGF-1 receptor activation, AKT activation can be induced, not only by PI3K-mediated phosphorylation of membrane lipid, but also via cAMP down-regulation owing to the fact that insulin/IGF-1 receptor activation increases cAMP phosphodiesterase activity [73]. AKT activation exacerbates UV-induced cell lethality via (NF-κB)-mediated amelioration of replication-driven DNA repair. Through phosphorylation of IKK (I kappaB kinase), the catalytic regulator of IκBα (nuclear factor of kappa light polypeptide gene enhancer in B-cells inhibitor, alpha), AKT mediates NF-κB activation in association with mTOR and Raptor [74,75]. Phosphorylated p65 and p50 subunits of NF-κB then translocate to the nucleus to bind to the promoter of FANCD2 (Fanconi anemia group D2 protein). The binding NF-κB suppresses FANCD2 transcription and leads to defective DNA repair when FANCD2 monoubiquitination is insufficient for forming complexes at the DNA damage loci that precede the repair of DNA double stranded break during DNA replication and transcription [76,77]. 

In addition to the cellular metabolic switch in response to UV-induced TKR activity, UV-induced mitogenic activity ameliorates the DNA damage responses of cells that express light-stimulated receptor kinases via activation of the mTOR-(NF-κB)-FANCD2 pathway. Evidence has been obtained that skin melanoma cells display greater UV-induced mitochondrial oxidative stress under mTOR inactivation, implying mTOR promotes glycolysis for the maintenance of energy and redox homeostasis [18]. Combined with the observation of UVR-induced increase in the S phase population, UVR-mediated mitogenic effects on skin melanoma cells is hypothesized to compromise the DNA repair capacity of cells during DNA synthesis [18]. This implies that receptor signaling modulates cell sensitivity to UV in association with mTOR activity [78]. 

## 3. Kinase Activation by UA Modulates UV-Induced Oxidative DNA Damage

UV radiation not only stimulates cells by increasing TKR activation, but also damages cells by introducing reactive oxygen species (ROS) and inducing protein and DNA adducts via photochemical reactions [79,80,81,82]. ROS-triggered PI3K activation can transduce oncogenic AKT-mTOR signaling; mTOR can increase the phosphorylation of both eukaryotic initiation factor 4E-binding protein1 (4EBP-1) and S6K1, leading to the translation and expression of mRNAs encoding several major anti-apoptotic proteins including XIAP, c-IAP1, Bcl-XL, and BCl-2 [83]. Through the integration of several upstream signals, including growth factors, nutrients, energy levels, and stresses, mTOR is differentially regulated by TSC2 via phosphorylation [84]. Studies have shown that the phosphorylation sites of TSC2 at Ser981, Ser1130 and Ser1132 by AKT impair the ability of TSC2 to inhibit the activator of mTOR, GTP-bound Rheb (Ras homolog enriched in brain) [84], whereas TSC2 phosphorylation at Thr1271 and Ser1387 by AMPK inhibits mTORC1 activity in an ATM-dependent manner [85]. In addition to those actions, AKT indirectly activates mTOR through inhibition of TSC2, while mTORC2 also responds to UV by forming into a complex with DNA-PKcs to phosphorylate AKT at Ser-473 [86]. Through its activation by phopshorylation, AKT suppresses homologous recombination repair (HHR) via TopBP1 (DNA topoisomerase 2-binding protein 1) [87]. Functioning as an adaptor protein for ATR, TopBP1 facilitates Chk1 phosphorylation as a consequence of cAMP-mediated AKT inactivation via PKA-dependent Rap1b phosphorylation [83,84,85,86,87,88]; however, the phosphorylation of TopBP1 at Ser-1159 by AKT induces TopBP1 oligomerization under oxidative stress and thereby prevents its recruitment to chromatin and ATR binding sites [89]. 

Recent studies have demonstrated the modulatory effects of phytochemicals on UV-induced ROS and DNA damage. For instance, many flavonoids present in red wine, cocoa and tea absorb UV light and hence reduce radiation-induced photodamage and photocarconogenesis [90]. Natural compounds from broccoli and the Australian dessert tree *Acacia victoriae* exhibit antioxidant activity against UV-induced oxidative stress through activation of phase II detoxification enzymes and antioxidant proteins via the Nrf2 (nuclear factor erythroid-derived 2-related factor 2)/ARE (antioxidant response element) system. In addition, phytochemicals, such as extracts of soybean and rhubarb, can regulate the activity of protein tyrosine kinase to elicit hormonal actions on human systems [90,91]. Phytochemical-induced down-regulation of tyrosine kinase activity decreases UV-stimulated production of pro-inflammatory enzymes and cytokines, including cyclooxygenase-2, prostaglandin, TNF-α (tumor necrosis factor α), and IL-1α (interleukin-1α) [92,93,94,95]. The natural pentacyclic triterpenoid, UA, which is found in several edible plants, exhibits anti-oxidant and anti-inflammatory properties partly via insulin receptor signaling [96]. Administration of UA to mice suppresses ischemic injury induced by cerebral artery occlusion through Nrf2 activation, which reduces the malonyldialdehyde formed as an end product of lipid peroxidation, along with NF-κB and TLR4 expression. Based on* in vitro* cell studies, UA decreased UVB-induced lipid peroxidation, oxidative DNA damage and cytotoxicity through both enzymatic activity modulation and signal transduction [23,97]. It has been shown that UA scavenges free radicals through up-regulation of superoxide dismutase and catalase expression in a dose-dependent and bioavailable manner [23,98,99]. On the other hand, UA-induced AKT activation in C2C12 skeletal myotubes is found to be elicited by ligand-dependent activation of the insulin receptor or the IGF-1 receptor [100]. UA increases mRNA levels of HK2 (hexokinase 2) and IGF-1, leading to enhanced glucose utilization and (IGF-1)-AKT signaling. Furthermore, UA enhances glucose uptake by inhibiting T-cell protein tyrosine phosphatase and src homology phosphatase-2 for sustaining insulin/IGF-1 receptor phosphorylation [101,102]. Interestingly, the increase in IGF-1 expression by UA increases insulin-induced AKT phosphorylation which promotes anabolic protein synthesis in skeletal muscles, but not adipose tissues [103,104]. These findings reveal the potential of UA to enhance photosensitization via facilitating IGF-1 receptor-driven AKT activation.

### 3.1. UA-Induced p53 Activation and Modulation of UV-Invoked ROS

AKT mediates p53 degradation via MDM2 activation as well as the inhibitory phosphorylation of glycogen synthase kinase 3β (GSK3β). That is to say, p53-activated DNA repair can be abrogated by AKT signaling, yet AKT-enhanced mTOR activity supports p53-mediated antioxidant defense upon irradiation [105,106]. Under moderate p53 expression, Nrf2 is up-regulated and activated by p21^Cip1^, allowing transactivation of downstream antioxidant genes that contain an antioxidant response element (ARE) in the promoter regions [107,108]. As a downstream effecter of p53, p21^Cip1^ associates with the DLG motif of Nrf2 and disrupts the binding between Keap1 and Nrf2, leading to inactivation of Cullin-3-based ubiquitin E3 ligase for Nrf2 [109,110]. Nrf2 stabilization thereby induces the expression of NAD(P)H: quinone oxidoreductase 1 (NQO1), glutathione biosynthetic enzymes (glutathione cysteine ligase modifier subunit and glutathione cysteine ligase catalytic subunit), and GSH-dependent antioxidant enzymes (glutathione peroxidase 2 and glutathione S-transferases), which scavenges superoxide via NADH and NADPH oxidation in response to UV irradiation [110,111,112,113]. Nevertheless, the nuclear translocation of Nrf2 can be inhibited by GSK3β via mTOR inactivation, indicating that cells relay aberrant mitogenic activation to PIKK family members for eliciting antioxidant defense against UV-induced DNA adducts [106,107]. 

**Figure 2:**
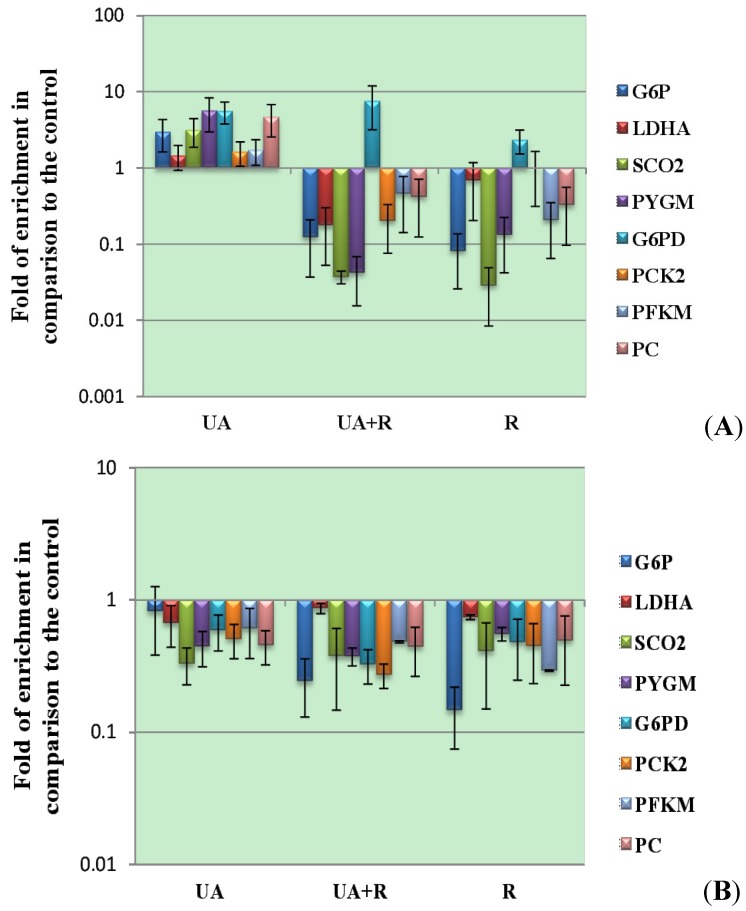
Transcriptional modulation of genes involved in glucose metabolism by UA in response to UVR. Transcription levels of glucogenetic genes in RPE cells (Panel A) indicate that the transcription of glycolytic and glucogenetic genes, except for G6PD in RPE cells, was down-regulated by UVR. The up-regulation of G6PD by UVR was further augmented by the pretreatment with UA. On the other hand, all investigated genes in SM cells were down-regulated by UA and/or UVR (Panel B). Cell culture and treatment was described in Reference 18. RNA were extracted from cells in control, UA-treated (denoted as UA), UA-plus-UVR-treated (denoted as UA+R), and UVR-treated (denoted as R) groups with TRIZOL reagent, followed by PCR analysis conducted with ReadyMix Taq PCR Reaction Mix with MgCl_2_ (Sigma-Aldrich, St. Louis, MO, USA) on Thermocycler (7900 HT Fast Real-Time PCR System, Applied Biosystems, Foster City, CA, USA). Quantitative results represent the mean values and standard errors of triplicate measurements. Two-way ANOVA was applied for analyzing the influence of UA or/and UVR on the transcription of investigated genes. G6P: a gene coding for glucose-6-phosphatase; LDHA: a gene coding for lactate dehydrogenase A; SCO2: a gene coding for synthesis of cytochrome c oxidase, subunit 2; PYGM: a gene coding for the muscle isoform of glycogen phosphorylase; G6PD: a gene coding for glucose-6-phosphate dehydrogenase; PCK2: a gene coding for phosphoenolpyruvate carboxykinase 2 (mitochondria); PFKM: a gene coding for the muscle isoform of phosphofructokinase; PC: a gene coding for pyruvate carboxylase.

Previously, we have observed that UVR induced mild p53 activation led to the up-regulation of glucose-6-phosphate dehydrogenase (G6PD) at the mRNA level, presumably through activation of Nrf2 in orchestration with mTOR activation, in RPE cells (Figure 2) [18,114,115]. The UVR-induced G6PD up-regulation was further promoted by UA pretreatment, leading to alleviation of UVR-induced mitochondrial oxidative stress and oxidative DNA damage [18,116]. In contrast, UVR suppressed the transcription of G6PD, and UA pretreatment further down-regulated G6PD transcription in skin melanoma cells. Mechanistically, mTOR inhibition eliminated UVR-induced p53 activation in both cell types, while differentially regulating p53 activation upon UA treatment. These results suggest cell-dependent responses to mTOR inhibition upon UA-mediated regulation of p53 activation. UA agonizes signaling of insulin/IGF-1 receptor and elicits AMPK-mediated p53 activation, which is abolished by mTOR-mediated IRS-1 degradation downstream of IGF-1 receptor signaling [117]. Intriguingly, mTOR inhibition did not up-regulate UA-induced p53 activation in skin melanoma cells. Moreover, mTOR inhibition mitigated p53 activation induced by UA-and-UVR combined treatment, in both RPE and skin melanoma cells, but specifically sustained the NF-κB activation in the latter cell line [18]. These phenomena suggest crosstalk between UVR- and UA- induced signaling, reflecting the fundamental metabolic disparity between normal and malignant cells linked to their differences in aerobic glycolysis described by Dr. Otto Heinrich Warburg in 1924. The Warburg effect depicts the exclusive expression of pyruvate kinase M2 in cancer cells to generate ATP quickly, *versus* mitochondrial oxidative phosphorylation by normal cells [63]. As the mitogenic action through the AKT-mTOR pathway propels aerobic glycolysis more in skin melanoma cells than in RPE cells, the UVR- or UA-induced p53 activation can result in tumor glycolysis inhibition, leading to a metabolic switch from lactate fermentation to pyruvate oxidation, preferentially occurring in skin melanoma cells [18,118]. Correspondingly, UVR-induced mitochondrial metabolism in skin melanoma cells was enhanced by pretreatment with UA. In comparison, mitochondrial metabolism in RPE cells was not promoted by UA due to the greater activation of p53 versus NF-κB activation (following the mechanism illustrated in the next paragraph) even though our real-time PCR analysis indicates that UA mediated SCO2 (synthesis of cytochrome c oxidase 2) transcription upon p53 activation (Figure 2) [18,119].

**Figure 3:**
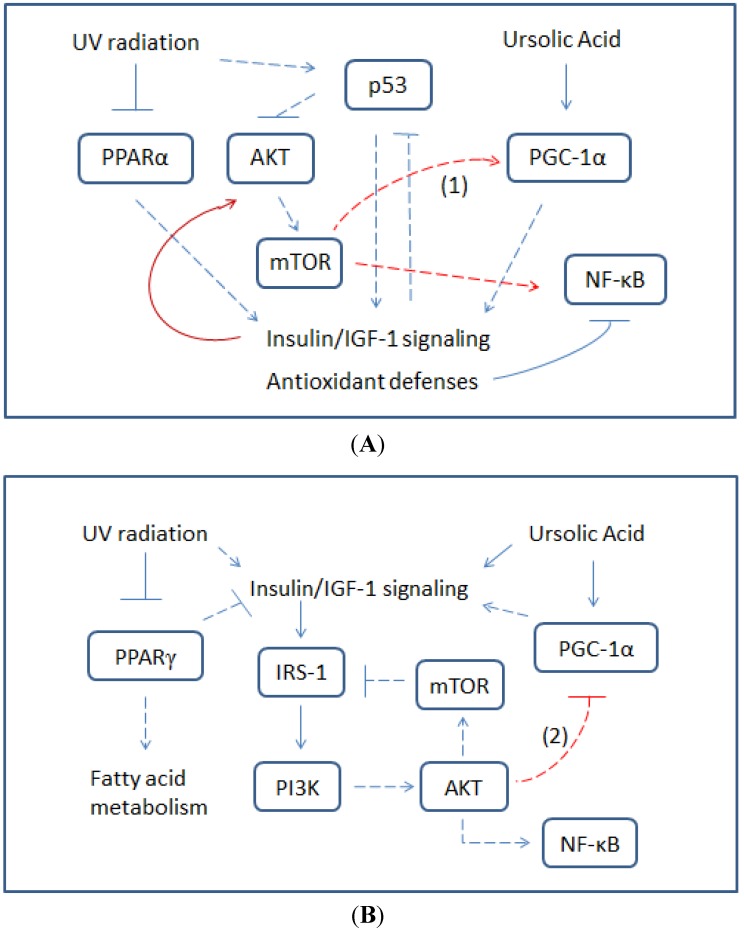
Schematic descriptions of crosstalk between UV- and UA-induced cell signaling and effects. In RPE cells, UV activates p53 which up-regulates IGF-1 signaling without affecting NF-κB activation (**A**). Upon (IGF-1)-provoked AKT-mTOR signaling, mTOR phosphorylates NF-kB but also mediates inactivation of NF-kB via PGC-1α stabilization. Empirically, the PIKK family member, mTOR, can enhance IGF-1 signaling via suppression of GSK3 activity. In skin melanoma cells, UV induces phosphorylation of p38MAPK and AKT to inactivate PPARγ and inhibit PGC-1α transcription, leading to the up-regulation and down-regulation of the IGF-1 receptor, respectively (**B**). Upon UA-mediated AMPK activation, PGC-1α can be up-regulated to promote mitochondrial oxidation as well as IGF-1 receptor expression. Notes for mitochondrial redox signaling: (1) AKT prevents PGC-1α degradation thus promoting UA-mediated antioxidant defenses via mTOR-suppressed GSK3 activity [123]; (2) AKT inhibits PGC-1α transcription and counteracts UA-mediated mitochondrial biogenesis by phosphorylating and promoting the nuclear exclusion of FoxO1. Dashed lines represent indirect mechanisms.

UVR-induced NF-κB was first shown to be specifically down-regulated by UA and/or mTOR inhibition in RPE cells, indicating differential mTOR signaling under UA-mediated regulatory effects on NF-κB activation. We speculate that the UVR-mediated PI3K-AKT-mTOR pathway and p38 MAPK-MAPKAPK2 (MAP kinase-activated protein kinase 2)-mTOR is responsible for NF-κB activation, by which the nuclear translocation of RelA/p65 can transactivate p53 promoter to result in enhanced mitochondrial oxidation [18,120,121]. The up-regulated p53 has been shown to interfere with the interaction between mortalin (a heat shock 70 family member) and RelA. Under p53 interference, Mortalin fails to sequester RelA into mitochondria to suppress mitochondrial gene transcription, leading to the promotion of oxidative phosphorylation [122]. In regard to the finding that UA and/or mTOR inhibition differentially modulated UVR-induced NF-κB activation and mitochondrial oxidative stress in skin melanoma* versus* RPE cells, we speculated that NF-κB activation of RPE cells is decreased through UA-mediated insulin/IGF-1-dependent anabolic metabolism against mTOR activity, while the hyperactive tyrosine kinase of IGF-1 receptor in skin melanoma cells manifests NF-κB activation and mitochondrial oxidation via the PI3K-AKT cascade (Figure 3) [18]. 

### 3.2. Modulatory Effects of UA on Cellular Response to UV-Induced TKR Down-Regulation

Although IGF-1 receptor activity can be maintained by UV-mediated inhibition of PTPs, *igf-1* transactivation can be inhibited by UV-mediated down-regulation of PPARα, leading to reduced mitochondrial oxidation [52,53]. The down-regulated IGF-1 signaling is expected to result in p53 stabilization and subsequent NF-κB inhibition via inactivation of PI3K in RPE cells. Comparatively, UA-mediated AMPK signaling promotes the cytoplasmic translocation of glucose transporters and increases the expression of the PPAR-γ coactivator 1α (PGC-1α), thus, up-regulating insulin signaling and mitochondrial biogenetics [124,125]. By interacting with PPAR-γ, PGC-1α can regulate the activity of the nuclear respiratory factors, Nrf1 and Nrf2, for expression and function of the respiratory chain and cytochrome oxidase in mitochondrial biogenesis [126,127,128]. Several genes including gluconeogenetic enzymes (glucose-6-phosphatase and phosphoenolpyruvate carboxykinase), glycolytic enzymes (phosphofructokinase and lactate dehydrogenase A), and enzymes involved in oxidative phosphorylation and glycogenolysis (cytochrome c oxidase and glycogen phosphorylase) are capable of being indirectly regulated by PGC-1α (Table 1) [129,130,131,132,133].

**Table 1:** Transcription of metabolic genes regulated by PGC-1 α.

Enzymes	mRNA Expression	Cell Lines, Tissues or Organs Invetigated
Glucose-6-phosphatase	Up-regulation	Human hepatic carcinoma and mouse liver [127,129]
Phosphoenolpyruvate carboxykinase	Up-regulation	Human hepatic carcinoma [129]
Phosphofructokinase	Down-regulation	Mouse skeletal muscle [128]
Lactate dehydrogenase A	Down-regulation	Mouse skeletal muscle [127]
Cytochrome c oxidase	Up-regulation	Human kidney [128]
Glycogen phosphorylase	Down-regulation	Mouse skeletal muscle [128]

To determine whether UA-mediated tumor sensitization and photoprotection of normal cells are regulated by differential energy metabolism, the expression of multiple genes involved in glucose metabolism has been studied. The transcription of investigated genes was promoted by UA in RPE cells but antagonized in skin melanoma cells, indicating that UA-mediated AMPK-(PGC-1α) signaling enhances metabolic gene transcription in RPE cells while inhibiting the transcription in skin melanoma cells by eliciting AKT-inactivated PGC-1α expression, as indicated by the downstream effecter, NF-κB (Figure 3) [18,124]. Studies have shown that PGC-1α transcription can be down-regulated by AKT-mediated nuclear exclusion of FoxO1, while its proteosomal degradation can be elicited by GSK3β upon mTOR-mediated phosphorylation [125]. The UA-induced NF-κB activation in skin melanoma cells was relatively sustained in response to mTOR inhibition, suggesting that both AKT and the AKT-activated mTOR exert regulatory effects on PGC-1α expression. The antagonistic effects of AKT and mTOR provide a potential explanation for the up-regulated transcription of metabolic enzymes observed in RPE cells but not in skin melanoma cells. As cytochrome c-oxidase activity is up-regulated by PGC-1α along with increase of cAMP response element-binding protein (CREB) for the transactivation capacity of Nrf1 and Nrf2 [134], UA-induced energy metabolism and phase II enzyme expression in RPE cells are proposed as defenses of normal cells against UV-induced energy depletion and oxidative stress.

**Figure 4:**
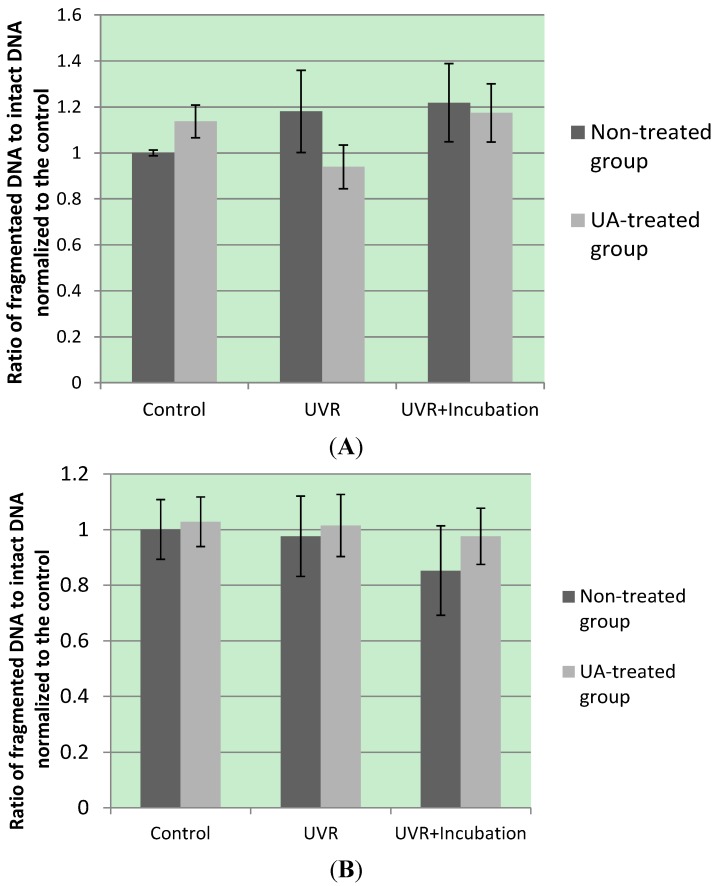
Detection of nuclear DNA fragmentation under UVR irradiation. Following irradiation (irradiation method was described in Ref. [18]), cells were directly harvested or incubated for another 30 minutes prior to harvesting. DNA fragmentation (assayed with agarose gel electrophoresis following a protocol adapted from that of Paola Bossù [137]) was differentially induced by UA and/or radiation in RPE (Panel (**A**)) and SM cells (Panel (**B**)). Gel imaging and analysis were conducted with a UV light box (ChemiDoc XRS system, Universal Hood with Camera, Bio-Rad) and the graphics software GIMP. Quantitative results represent the mean values and standard deviations of triplicate measurements and were analyzed with two-way ANOVA. Control: cells without irradiation; UVR: cells exposed to 10-min UV-VIS radiation; UVR+Incubation: cells were incubated 30-min after 10-min UV-VIS irradiation.

On the other hand, we speculate that AKT-mediated PGC-1α inhibition and the subsequent inactivation of metabolic gene transcription are more effectively exerted by skin melanoma cells to counteract mTOR-inhibited PGC-1α degradation upon UA-induced IGF-1 receptor signaling (Figure 3). This allows UA to preferentially sensitize skin melanoma cells to UVR,* i.e.*, by energy deprivation. Under UA-mediated energy deprivation, UVR-attenuated cAMP can be further restricted to mediate p53 proteosomal degradation via AKT-dependent p53-MDM2 interaction, leading to apoptosis of p53-reactive tumor cells [18,135]. By treating cells with UA, we found induction of DNA breakage in both RPE and skin melanoma cells (Figure 4). However, UA pretreatment decreased UV-induced DNA double-stranded breaks (DSBs) in RPE cells while potentiating UV-induced DNA DSBs and impairing DNA repair in skin melanoma cells. This result suggests activation of the (PGC-1α)-Nrf2 pathway, as well as inhibitory phosphorylation of insulin receptor substrates by mTOR signaling, strengthened cellular antioxidant defenses and attenuated AKT-suppressed HRR in RPE cells [136]. Nevertheless, (DNA-PK)-dependent NHEJ repair can be down-regulated by UA-mediated AMPK-p53 signaling and the consequent inhibition of AKT-mediated DNA-PK phosphorylation. As a result, UA modulates UVR-induced DNA damage responses in a cell-specific fashion, which is highly related to the resultant alteration of metabolism.

The abrogation of AKT activation by cAMP-PKA-Rap1b signaling reflects the important role of intracellular cAMP on the overall cell responses to UV-induced insults [138]. Because cAMP can be regulated by UV-activated adenylyl cyclase as well as IGF-1 receptor-mediated activation of cAMP phosphodiesterase, it provides a link between UA-induced photosensitivity and UA-abrogated DNA damage checkpoint and repair. UA affects the size of the intracellular cAMP pool in association with the signal transduction from IGF-1 receptor to PGC-1α through AKT activation (Figure 3) [100]. Inhibition of PGC-1α activity additionally leads to insufficiency of cellular antioxidant defense and deficiency of cellular energy metabolism against UV-induced DNA damage [14]. Under mild AKT activation, UA stimulates lipolysis by promoting HSL (hormone-sensitive lipase) translocation from cytosol to the lipid droplets via the cAMP-dependent PKA axis [139,140]. Lipolytic products, in turn, elevate intracellular cAMP, which can block ATM- and (DNA-PKcs)-mediated p53 activation and apoptotic signaling following UV-induced DNA damage [141,142]. 

## 4. Conclusions

Ultraviolet is a proven human carcinogen, which induces DNA lesions, including a (6-4) photoproduct [T(6-4)T] and a *cis-syn* cyclobutane TT dimer (T=T), and promotes transversion mutations [143]. In the United States, one in every five Americans develops skin cancer in the course of a lifetime. Up to 86% of melanomas have been hypothesized to result from solar UV exposure [144]. Additionally, more than 50% Americans have a cataract by the age of 80. With respect to the risk of UV-induced photocarcinogenesis and photodamage, the antitumoral and antioxidant functions of natural compounds may be useful for modulating UV-induced effects. When cells are exposed to UV, the induced DNA damage and oxidative stress provoke activation of PIKKs, PI3K and MAPK, leading to cell cycle arrest, DNA repair and metabolic adaptation via p53 and NF-κB signaling. By pretreating skin melanoma cells with UA, we observed that UVR-induced mitochondrial metabolic stress further increased with activation of NF-κB and p53, effects which were not found in RPE cells. This suggests a metabolic switch induced by UA occurred in the glycolytic cancer cells, causing severe oxidative stress and rendering cells apoptotic following UV irradiation. On the other hand, UA-mediated up-regulation of multiple enzymatic genes of glucose metabolism was specifically observed in RPE cells, while the lack of these responses in skin melanoma cells indicated the existence of a negative feedback loop of AKT activation to suppress PGC-1α expression in malignant cells. 

As many malignant cancer cell lines encompass over-expressed 3-phosphoinositide-dependent kinase 1 or a loss of PTEN function, the anabolism activator, insulin or its mimetics, usually exacerbate AKT-mediated PGC-1α suppression and reduce mitochondrial metabolism and antioxidant defenses [145,146]. Thereafter, energy starvation as well as UV-induced oxidative stress induces AMPK-mediated metabolic adaptation through inhibition of acetyl-CoA carboxylase and TORC1, along with p53 activation [147,148,149,150,151]. When p53 initiates mitochondrial bioenergenesis in the absence of mTOR activity, metabolic stress rapidly accumulates during the TCA cycle to perturb bioenergetic and redox homeostasis. As a result, lack of mTOR-mediated GSK3β inhibition for Nrf2-driven transactivation of antioxidant and detoxification genes can lead to cytochrome c release from mitochondria and putative induction of apoptosis.

UA modulates cellular sensitivity to UV by agonizing insulin-/(IGF-1)-mediated anabolism and up-regulating (PGC-1α)-mediated catabolism. Insulin-/(IGF-1)-mediated anabolism reduces intracellular cAMP, which in contrast is increased by (PGC-1α)-mediated catabolism. Differential expression of insulin/IGF-1 receptors in different cell lines, therefore, controls the intracellular cAMP pool and affects cellular responses to UV [152]. Through activation of adenylyl cyclase and ATM, UV increases intracellular cAMP and induces gluconeogenesis and mitochondrial oxidation for producing cellular antioxidant reductants from the PPP and TCA cycle. Additionally, cAMP reinforces homologous recombination repair by attenuating p53 accumulation and inhibiting AKT activity upon UV-induced DNA damage. The cAMP signaling, however, is negatively affected by insulin-/(IGF-1)-mediated cAMP reduction. As a result, the insulin mimetic, UA, can preferentially sensitize insulin-resistant cells toward UV-induced oxidative DNA damage. The metabolic and mitogenic effects of UA trigger a decrease in carbohydrate sources, leading to reduction of mTOR activity and activation of the AMPK-p53 axis. The UA-induced metabolic alterations predispose insulin-resistant cells to suffer a lack of energy and perturbation of redox homeostasis as the crosstalk between p53 and mTOR is diminished. In comparison, cells that are sensitive to insulin remain viable throughout mTOR inhibition due to the prevalence of oxidative metabolism for redox and energy homeostasis. The perspective presented by this review introduces a new understanding of the mechanism by which UA exerts differential effects on cells exercising different metabolic pathways in response to UV-induced oxidative stress and DNA damage.

## Abbreviations

ATM: ataxia telangiectasia mutated
AMPK: AMP-activated protein kinase
cAMP: cyclic adenosine monophosphate
CPD: cyclobutane pyrimidine dimers
DNA-PK: DNA-dependent protein kinase
G6PD: glucose-6-phosphate dehydrogenase
GSK3β: glycogen synthase kinase 3β
HRR: homologous recombination repair
IGF-1: insulin-like growth factor 1
IRS-1: insulin receptor substrate 1
MAPK: mitogen-activated protein kinase
mTOR: mammalian target of rapamycin
NF-κB: nuclear factor kappa-light-chain-enhancer of activated B cells
NHEJ: non-homologous end-joining
Nrf2: nuclear factor erythroid-derived 2-related factor 2
PGC-1α: PPAR-γ coactivator 1α
PIKK: phosphatidylinositol 3-kinase-related kinase
PKA: cAMP-dependent protein kinase
PPAR: peroxisome proliferator-activated receptor
PPP: pentose phosphate pathway
redox: reduction-oxidation
ROS: reactive oxygen species
RPE: retinal pigment epithelium
TKR: tyrosine kinase-coupled receptor
UA: ursolic acid
UV: ultraviolet
UV-VIS: (or UVR) radiation with spectrum wavelength ranging from UV to visible light
